# Determinants of Bacterial Morphology: From Fundamentals to Possibilities for Antimicrobial Targeting

**DOI:** 10.3389/fmicb.2017.01264

**Published:** 2017-07-10

**Authors:** Muriel C. F. van Teeseling, Miguel A. de Pedro, Felipe Cava

**Affiliations:** ^1^Laboratory for Molecular Infection Medicine Sweden, Department of Molecular Biology, Umeå Centre for Microbial Research, Umeå University Umeå, Sweden; ^2^Centro de Biología Molecular “Severo Ochoa” – Consejo Superior de Investigaciones Científicas, Universidad Autónoma de Madrid Madrid, Spain

**Keywords:** bacterial morphology, peptidoglycan, cytoskeleton, antimicrobials, cell shape inhibitors

## Abstract

Bacterial morphology is extremely diverse. Specific shapes are the consequence of adaptive pressures optimizing bacterial fitness. Shape affects critical biological functions, including nutrient acquisition, motility, dispersion, stress resistance and interactions with other organisms. Although the characteristic shape of a bacterial species remains unchanged for vast numbers of generations, periodical variations occur throughout the cell (division) and life cycles, and these variations can be influenced by environmental conditions. Bacterial morphology is ultimately dictated by the net-like peptidoglycan (PG) sacculus. The species-specific shape of the PG sacculus at any time in the cell cycle is the product of multiple determinants. Some morphological determinants act as a cytoskeleton to guide biosynthetic complexes spatiotemporally, whereas others modify the PG sacculus after biosynthesis. Accumulating evidence supports critical roles of morphogenetic processes in bacteria-host interactions, including pathogenesis. Here, we review the molecular determinants underlying morphology, discuss the evidence linking bacterial morphology to niche adaptation and pathogenesis, and examine the potential of morphological determinants as antimicrobial targets.

## Introduction

**FIGURE 1:**
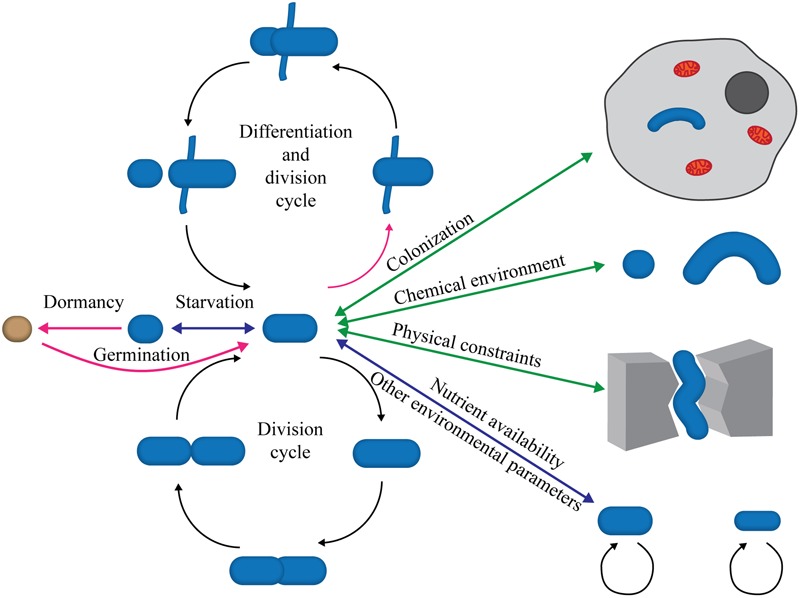
Morphological plasticity and the bacterial life cycle. The scheme illustrates the continuous modulations affecting bacterial shape throughout the life cycle. These changes can be either cyclic (division cycles) or sporadic in response to changing conditions, the presence of chemicals, colonization of other organisms or environments, nutrient depletion or abundance, etc. Most shape alterations are reversible (double-headed arrows) and could be considered adaptive phenomena, whereas others are irreversible (single-headed magenta arrows) and represent bona fide morphological differentiation processes, such as sporulation (orange sphere) or polymorphic cell cycles.

The variation of bacterial cell shapes is often underappreciated. In addition to the well-known rods and cocci, more exotic shapes such as stars, mustaches, serpentines, and branches represent a large, although undefined, proportion (Young, 2006; Kysela et al., 2016). The characteristic morphology of a bacterial species is maintained through countless generations but is periodically modified within set limits during bacterial division and life cycles (**Figure 1**). Bacterial shape is genetically determined, but physical forces (internal and external) exerted on cells are increasingly recognized as major players in morphogenesis. To ensure constant bacterial morphology over generations despite these forces, shape maintenance must be an active process guided by robust regulatory circuits. This is evidenced by the development of aberrant morphology upon mutations. Shape dictates the interactions between a bacterial cell and its environment, most notably small-molecule traffic (via the surface/volume ratio), motility, formation of multicellular aggregates, habitat colonization (including eukaryotic hosts and consequently pathogenesis and symbiosis), predation, and resistance (see Young, 2006, for a comprehensive review). Therefore, morphogenesis should be viewed as a major evolutionary and adaptive process that contributes greatly to prokaryotic ubiquity and versatility.

Bacterial shape is primarily dictated by the peptidoglycan (PG) sacculus (Salton and Horne, 1951; Weidel et al., 1960), a polymeric macromolecular structure that surrounds the cytoplasmic membrane and is the only “solid” element in the bacterial envelope. PG is present in essentially all bacteria [the number of exceptions is quickly dwindling as better detection methods are developed (Pilhofer et al., 2013; Liechti et al., 2014; Jeske et al., 2015; van Teeseling et al., 2015; Rast et al., 2017)] and wraps the cytoplasmic membrane like an elastic net (de Pedro and Cava, 2015). PG is a polymer of glycan chains crosslinked by peptides. The structure of the monomeric subunit, *N*-acetyl-glucosaminyl-*N*-acetyl-muramyl-L-alanyl-D-glutaminyl-L-(meso)diaminopimelyl-D-alanyl-D-alanine (GlcNAc-MurNAc-L-Ala-D-Glu-L-mesoDAP-D-Ala-D-Ala), is remarkably conserved throughout the bacterial phylogenetic tree. The few variations are either a change in the amino acid sequence of the stem peptide (almost always the di-amino acid at position 3) or the consequence of accessory reactions that modify the basic subunit (e.g., O-acetylation of sugars or amidation of dicarboxylic amino acids) (Vollmer, 2008; Cava and de Pedro, 2014).

The precursors for PG biosynthesis, uridine diphosphate-*N*-acetylglucosamine (UDP-GlcNAc) and UDP-MurNAc-pentapeptide, are synthesized in the cytoplasm by the enzymes MurA-F (Barreteau et al., 2008). The enzyme MraY couples the UDP-MurNAc-pentapeptide to undecaprenyl phosphate to produce the membrane-anchored lipid I (Manat et al., 2014). Subsequent addition of GlcNAc to lipid I by MurG results in inward-oriented lipid II molecules. Translocation to the outer face of the cytoplasmic membrane is performed by the flippase MurJ (Sham et al., 2014), with the likely participation in some species of AmiJ (Meeske et al., 2015) and the SEDS (shape, elongation, division and sporulation) proteins RodA and FtsW (Mohammadi et al., 2011; Scheffers and Tol, 2015; Leclercq et al., 2017). Once transferred to the external side of the cytoplasmic membrane, the GlcNAc-MurNAc-pentapeptide moiety of lipid II becomes accessible to enzymes with glycosyltransferase (GT) and transpeptidase (TP) activities, which catalyze linear polymerization and peptide crosslinking, respectively. The undecaprenyl diphosphate released in the polymerization reaction is flipped back, dephosphorylated and reused for the cyclical transport of new precursors (Manat et al., 2014). Bifunctional proteins with GT and TP activities are universal and concurrent with monofunctional representatives of both activities. The SEDS protein RodA was recently identified as a novel GT enzyme *in Bacillus subtilis*, and seems to play the same role *in Escherichia coli* (Cho et al., 2016; Meeske et al., 2016). A ubiquitous class of enzymes involved in crosslinking is DD-transpeptidases, which are inhibited by covalent binding of beta-lactams and accordingly were first identified as penicillin-binding proteins (PBPs) (Sauvage et al., 2008). In mature PG, D,D-crosslinks between the D-Ala at position 4 in the stem peptide of one subunit and the di-amino acid at position 3 (either directly or through intermediate peptides) of a nearby stem peptide are universal. Additional crosslinking mechanisms involving specific sets of enzymes and distinctive stereochemistry are relatively common (Vollmer et al., 2008a).

As the PG layer is a covalently closed structure, the addition of new material requires concomitant cleavage of pre-existing bonds by PG hydrolases to permit enlargement of the sacculus. PG remodeling and maturation are mostly mediated by PG hydrolases (Vollmer et al., 2008b). As a group, these enzymes target every bond (glycosidic and peptidic) sustaining the PG fabric. Organisms can encode many hydrolases, which are often redundant (35 and counting in *E. coli*) (van Heijenoort, 2011). In addition to the enlargement process, the sacculus is subject to a complex and dynamic metabolism involving a large number of proteins that are not directly involved in the integration of precursors. Modifications related to subunit aging, growth state, nutritional conditions, population density, and stress response have been reported (Cava and de Pedro, 2014). Of particular interest to the present work are modifications causing changes in shape and the differentiation of new structures during the cell and life cycles of bacteria.

In this review, we will discuss how sacculi are molded and altered to produce typical bacterial morphologies, primarily in Gram-negative bacteria. The proteins involved and the underlying mechanisms will be elaborated.

## Generation of Cell Shape

Because of its covalently closed, net-like structure, the PG sacculus retains a specific shape and imposes this shape on the bacterial cell body. Despite a certain degree of deformability due to the elastic nature of the PG fabric (Männik et al., 2009), isolated sacculi faithfully retain the shape of the corresponding cell. However, the sacculi themselves lack the inbuilt information and/or features to determine their precise shape. Coding of epigenetic structural information in the 3D organization of the molecule has been proposed (Turner et al., 2010). However, no hard evidence supports this hypothesis. Furthermore, the current view of the sacculus as a relatively disordered array (de Pedro and Cava, 2015) and the ability of “cell wall-less” forms to regenerate bacillary shapes (Billings et al., 2014; Kawai et al., 2014) argue against such coding. Even if a particular disposition of incoming new precursors might be favored by the pre-existing order (or lack thereof) of the older material, this does not necessarily indicate a global shape-defining role. Therefore, it seems reasonable to assume that while the sacculus is the element that confers and preserves a defined shape and size, the generation of that shape depends on the dynamics and topology of biosynthetic complexes rather than the sacculus itself.

The simple growth of a closed net subjected to cytoplasmic turgor pressure poses some critical constraints on the incorporation of new material to cause an effective enlargement. These constraints must be overcome by morphogenetic mechanisms. Simple attachment of incoming precursors to the sacculus would result only in thickening. Indeed, PG endopeptidases that permit expansion by cleaving existing crosslinked peptides have been identified in *E. coli, Vibrio cholerae*, and *B. subtilis* (Hashimoto et al., 2012; Singh et al., 2012; Dörr et al., 2013). However, if insertion of new material, and concomitant cleavage of old crosslinks, would happen constantly and evenly over the whole surface of the sacculus, this would lead to a homogeneous expansion of the growing structure. This mechanism by itself would not allow for the differentiation of new features. To generate shapes other than a sphere, incorporation must occur at distinct rates in different locations and for defined periods of time. Budding, for instance, would require a faster rate of precursor incorporation at the budding site than in the surrounding area. The morphogenetic process in bacteria not only requires physical enlargement, but also must allow periodic division events to increase the number of individuals.

As the mode of division of common model organisms, symmetrical binary fission is the best-known division mechanism and represents an elegant, intuitive mechanism to ensure shape conservation (Angert, 2005). However, alternative ways of division also occur (Angert, 2005). The only critical condition for division is the equitable distribution of both the genetic material and the biochemical components required to express the genetic potential. Division must be regulated in such a way that further divisions are not allowed before these conditions are fulfilled by the daughter cells. Many bacterial species divide by alternative mechanisms, often producing offspring cells that are quite dissimilar in size, shape and physiology from the mother cells (**Figure 1**). In these instances, the “juvenile” cells must undergo complex developmental programs to generate the characteristic morphology before committing to a subsequent round of division (e.g., Hirsch, 1974; Curtis and Brun, 2010; Williams et al., 2016; Cserti et al., 2017).

Cytokinesis implies the scission of the bacterial cell wall at genetically determined locations and cell cycle times while preserving cell integrity. The sacculus is a common substrate in cytokinesis and growth (enlargement and differentiation), which are mediated by closely related enzymatic complexes. As described below, the elements responsible for the dynamics and topology of PG biosynthetic complexes are slowly being unraveled, thanks to current advances in genetics and visualization techniques.

### Positioning and Guiding Peptidoglycan Synthesis: Cytoskeletal Elements

Since PG dictates bacterial cell shape, regulation of the location and timing of the synthesis and degradation of PG throughout the cell cycle is of key importance. Bacteria use cytoskeletal elements to position proteins involved in PG synthesis and hydrolysis in large, intricately regulated protein complexes. The cytoskeletal elements FtsZ and MreB are relatively conserved, but the exact composition of the protein complexes associated with FtsZ and MreB varies from species to species. Unless stated otherwise, we base our description on the model organism *E. coli.*

#### Actin-Like Cytoskeletal Elements

The actin-like cytoskeletal protein MreB influences bacterial morphogenesis by coordinating cell wall biosynthesis spatiotemporally (Carballido-López, 2007; Errington, 2015). The MreB protein is important for maintaining the rod shape in bacteria (Wachi et al., 1987; Doi et al., 1988; Levin et al., 1992), is conserved in many non-spherical bacteria (Jones et al., 2001), and forms actin-like filaments (Jones et al., 2001; van den Ent et al., 2001), thus motivating extensive studies of this protein. Multiple roles of MreB have been identified (Carballido-López, 2007; Busiek and Margolin, 2015), although directing PG insertion during elongation appears to be the main role of MreB in most organisms.

Although the exact localization pattern of MreB in bacterial cells has been highly debated (Errington, 2015), it is widely accepted that MreB forms membrane-bound patches or filaments in an ATP-dependent manner (Salje et al., 2011). Multiple studies show that these filaments move along the periphery of the cell (Errington, 2015). MreB interacts with numerous proteins involved in PG biosynthesis and hydrolysis, which are clustered in a large protein complex called the elongasome (Szwedziak and Löwe, 2013; Laddomada et al., 2016). These proteins include the PBPs PBP1A and PBP2; the hydrolase LytE (in *B. subtilis*) (Carballido-López et al., 2006); the enzymes DapI, MurC, MurD, MurE, MurF, MurG, and MraY, which are involved in the synthesis of lipid II; and the protein FtsW, for which functions as a flippase of lipid II (Mohammadi et al., 2011) and as a PG polymerase (Meeske et al., 2016) have been described.

The movement of MreB filaments along the membrane is correlated with active PG biosynthesis (Domínguez-Escobar et al., 2011; Garner et al., 2011; van Teeffelen et al., 2011; Cho et al., 2016). Originally, this movement was proposed to be caused by treadmilling of the MreB filaments (Soufo and Graumann, 2004; Kim et al., 2006). A revised hypothesis posited that the movement of the elongasome depends on PG synthesis, either by insertion of new glycan strands of PG by a bifunctional PBP in a pushing movement or by pulling of hydrolases degrading old PG strands (Errington, 2015). Recent studies in *E. coli* have shown that MreB filaments only move if RodA can polymerize the glycan backbone of PG, thereby demonstrating that polymerization by the SEDS protein RodA, and not bifunctional PBPs, drives MreB movement (Cho et al., 2016).

The combination of time-lapse microscopy with biophysical simulations has provided a deeper understanding of how MreB localization and subsequent cell wall synthesis lead to elongation of bacterial cells (Ursell et al., 2014). MreB localizes preferentially to negatively curved membrane regions, where it directs local cell growth by PG incorporation, leading to a more positive curvature. MreB then moves to another membrane region with negative curvature, where it stimulates PG insertion. Simulations show that this pattern of dynamic growth bursts in regions of negative cell curvature leads to a straight morphology. Recent results suggest that MreB also affects cell diameter, as the helical pitch angle of MreB filaments correlates with the diameter of the model organism *E. coli* (Ouzounov et al., 2016). These findings imply a sophisticated relationship between bacterial morphology and the structure and orientation of the MreB filaments with respect to the membrane.

Actin-like cytoskeletal elements other than MreB have been identified in bacteria (Carballido-López, 2007; Busiek and Margolin, 2015). The functions of only a few of the 35 known families of actin-like proteins have been studied (Derman et al., 2009). Of these, the membrane-associated, filament-forming FtsA is involved in the formation and function of the divisome (see below) in multiple bacteria (Pinho et al., 2013). There, the role of FtsA is hypothesized to be comparable to that of MreB (Szwedziak and Löwe, 2013). In this scenario, FtsA has a crucial role in guiding cell wall synthesis and remodeling during cell division. Several actin-like proteins, notably ParM (Jensen and Gerdes, 1997) and AlfA (Becker et al., 2006), are involved in DNA segregation.

#### Tubulin-Like Cytoskeletal Elements

The bacterial tubulin-homolog FtsZ is a key protein in cell division that is present in nearly all bacteria (Vaughan et al., 2004; Bernander and Ettema, 2010). FtsZ polymerizes into (proto)filaments that curve and thereby constrict the cytoplasmic membrane in a GTP hydrolysis-dependent fashion (Erickson et al., 1996; Li et al., 2013). During constriction, septal PG must be synthesized and/or the existing PG must be remodeled to create new poles for both daughter cells. The divisome, a protein complex associated with the ring formed by FtsZ (the Z ring), coordinates constriction and septal PG biosynthesis and remodeling (Haeusser and Margolin, 2016). Because FtsZ cannot bind the membrane, other proteins, such as the widely conserved actin homolog FtsA and ZipA, are required to tether FtsZ to the membrane. FtsA and ZipA are thought to affect the polymerization dynamics of FtsZ as well as the orientation of the protofilaments in the Z ring to contribute to proper Z ring function (Loose and Mitchison, 2014; Haeusser and Margolin, 2016). Different membrane anchors have been described in different species; the newly described anchor FzlC in *Caulobacter crescentus* has been shown to affect PG hydrolysis during cell division (Meier et al., 2016).

Multiple proteins involved in PG synthesis and remodeling are recruited to the divisome. In addition to PBP1B and PBP3, the divisome includes the flippase/PG synthase FtsW, PG hydrolases and hydrolase activators (van Heijenoort, 2011; Haeusser and Margolin, 2016). Multiple proteins in the divisome have been shown to directly or indirectly stimulate PG synthesis or hydrolysis. ATP hydrolysis by the ABC-like complex FtsEX, which interacts with FtsA, is required for PG synthesis, and FtsEX also plays a role in PG hydrolysis (Du et al., 2016). The protein FtsN, which binds directly to FtsA and PBP1B, stimulates septal PG synthesis (Müller et al., 2007). The function of PBP1B requires interaction with the lipoprotein LpoB (Paradis-Bleau et al., 2010; Typas et al., 2010). CpoB stimulates PBP1B in response to the state of the Tol-Pal system, which is responsible for constricting the outer membrane to ensure coordinated constriction of the cell envelope during cytokinesis (Gray et al., 2015). In *C. crescentus*, a flexible linker sequence inside FtsZ itself may be important for PG remodeling by affecting the degree of crosslinking and the length of the glycan chains (Sundararajan et al., 2015). Very recent studies have shown that the PG biosynthesis enzymes in the divisome synthesize PG at discrete sites that move around the cell division plane by treadmilling of FtsZ (Bisson-Filho et al., 2016; Yang X. et al., 2016).

Several studies indicate that FtsZ is not only important for PG biosynthesis during cell division but also contributes to sidewall synthesis, in a process known as preseptal PG synthesis. Preseptal PG synthesis has been described in *E. coli* (de Pedro et al., 1997) and *C. crescentus* (Aaron et al., 2007) and appears to be important during a larger part of the cell cycle in the latter. Many open questions remain, although two different mechanisms have been described for this preseptal PG incorporation in *E. coli*. The first mechanism requires the interaction of FtsZ with PBP2 (Varma et al., 2007; Varma and Young, 2009), a PBP that normally interacts with the elongasome instead of the divisome. In the second mechanism, FtsZ and ZipA, but not MreB and PBP2, are required for insertion of PG that appears to lack pentapeptides (Potluri et al., 2012). This mechanism is known as PIPS (PBP3-independent peptidoglycan synthesis). PIPS is thought to occur after elongation ends and before constriction of the cell begins (Potluri et al., 2012). Other studies suggest the occurrence of a different mechanism between elongation and division in *E. coli*. A direct interaction of MreB and FtsZ is crucial for proper cell division (Fenton and Gerdes, 2013), and the corresponding PBPs PBP2 and PBP3 colocalize and interact before division begins (van der Ploeg et al., 2013). These observations led to the hypothesis that at least part of the PG biosynthetic machinery might be transferred from MreB to FtsZ in preparation for cell division (Fenton and Gerdes, 2013). Further studies are needed to better understand which processes occur between elongation and division and how, if at all, the mechanisms described above are coordinated.

Bacterial tubulin-like proteins other than FtsZ exist (Busiek and Margolin, 2015). A function in DNA partitioning has been identified for several members of the TubZ family (Larsen et al., 2007), thus paralleling the function of some actin-like proteins (see above). Two other tubulin homologs, BtubA, and BtubB, have been identified in the phylum Verrucomicrobia (Jenkins et al., 2002). BtubAB forms filaments in the presence of GTP (Schlieper et al., 2005), but the function of these filaments remains unknown.

#### Intermediate-Like Cytoskeletal Elements

Bacterial intermediate filament (IF)-like structures are also involved in positioning the PG biosynthesis machinery. IF-like structures can polymerize into filaments or sheets, but in contrast to actin- and tubulin-like cytoskeletal structures, this polymerization occurs without binding and hydrolysis of nucleotides (Lin and Thanbichler, 2013). The three main classes of bacterial IF-like elements are bactofilins, coiled-coil-rich proteins (CCRPs) and cytoskeletal-like scaffolding proteins. In the domain Bacteria, IF-like proteins are widespread, and studies of several representative proteins support multiple roles, including morphogenesis, locomotion, cell division and intracellular localization of proteins.

The bactofilins BacA and BacB have a direct role in positioning the proteins involved in PG synthesis by localizing the bifunctional PBP PbpC to the base of the stalk in *C. crescentus* during the transition from swarmer to stalked cell (Kühn et al., 2010). PbpC contributes to elongation of the stalk (Kühn et al., 2010), although it might also contribute to PG biosynthesis at other cellular locations and interact with divisome proteins as well as with other bifunctional PBPs (Strobel et al., 2014). Although conclusive evidence is lacking, it has been hypothesized that the bactofilins CcmA in *Proteus mirabilis* and BacM in *Myxococcus xanthus* are also involved in recruiting and positioning cell wall biosynthesic proteins (Hay et al., 1999; Koch et al., 2011). In the helical bacterium *Helicobacter pylori*, a CcmA protein has been implicated in cell shape (Sycuro et al., 2010). However, whether CcmA forms a cytoskeleton in this bacterium and, if so, how this putative cytoskeleton is involved in helical cell shape remains unclear. Several other proteins necessary for helical morphology have been described, the majority of which are PG hydrolases (see below and Bonis et al., 2010; Sycuro et al., 2010, 2012, 2013). An inviting hypothesis is that the CcmA protein forms a cytoskeleton that is involved in positioning these hydrolases so that they modify the degree of PG crosslinking only at specific sites. A similar mechanism may occur in the helical *Campylobacter jejuni*, although the role of its CcmA homolog in morphology has not been established (Frirdich et al., 2012, 2014). We expect that follow-up studies of the function of bactofilins, which are present in many bacteria (Kühn et al., 2010), will reveal more examples of bactofilins as tethers for PG enzymes (potentially organized in protein complexes) to permit more complex morphologies.

The role of the CCRP crescentin, the protein responsible for the curvature of *C. crescentus* cells (Ausmees et al., 2003), in PG biosynthesis is less direct (Cabeen et al., 2009). According to the current model, the lining of the crescentin filament along the inner curvature of the cell provides a compressive force that results in a higher PG synthesis rate at the outer curvature than at the inner curvature of the cell (Cabeen et al., 2009). The involvement of CCRP filaments in morphology has also been reported for *H. pylori* (Waidner et al., 2009; Specht et al., 2011; Schätzle et al., 2015). However, whether these CCRPs influence PG biosynthesis and, if so, via which underlying mechanism remains unclear. A mechanism similar to that of CreS has been proposed for the recently discovered CCRP CrvA, which is responsible for the curved morphology of *V. cholerae* (Bartlett et al., 2017). CrvA self-assembles at the inner face of the cell curvature and asymmetrically patterns PG insertion, resulting in more insertions in the outer face than the inner face. Strikingly, however, CrvA localizes in the periplasm and therefore forms a periskeleton rather than a typical cytoskeleton.

DivIVA is the most-studied protein in the third class of IF-like elements, the cytoskeletal-like scaffolding elements. This protein is restricted to Gram-positive bacteria. In some of these bacteria, notably the actinomycetes, DivIVA activates and recruits PG biosynthetic enzymes to the cell pole to establish polar growth (Lin and Thanbichler, 2013). No hard evidence supports the ability of the Gram-negative (evolutionarily unrelated) variant PopZ to recruit PG biosynthetic enzymes. However, PopZ (Grangeon et al., 2015), one PBP with a transglycosylase activity and an L,D-transpeptidase (Cameron et al., 2014) all localize to the growing pole in *Agrobacterium tumefaciens*. As the involvement of other likely candidates in recruitment of the PG biosynthesis machinery to the growing pole has recently been excluded, PopZ could very well be involved in localizing PG biosynthesis in at least some Gram-negative bacteria (Howell and Brown, 2016).

The first Gram-negative IF-like structure was identified only in 2003 in the form of crescentin (Ausmees et al., 2003), but the involvement of multiple IF-like structures in positioning or guiding PG biosynthesis has been demonstrated in several cases. We expect that further research will establish positioning of PG biosynthesis and modification as one of the functions of IF-like structures. We envision that IF-like proteins might even tether protein complexes reminiscent of the elongasome and divisome for this purpose. In that case, all three classes of cytoskeletal elements would have a complementary cell wall synthesizing protein complex. The first few examples of proteins interacting with IF-like cytoskeletal elements suggest that these protein complexes might be involved in shape modification (especially when compared with the canonical coccoid and rod shapes). If this role were to be verified, we expect the compositions of these protein complexes to be more diverse and considerably less conserved than those of elongasome and divisome complexes, given the morphological diversity with which the IF-like cytoskeletal elements might be associated.

### Post-Insertional Modifications of the Sacculus

In addition to positioning of PG synthesis by cytoskeletal elements, enzymes that affect the chemical composition of the PG can impact cell shape. One of the first indications that PG hydrolytic enzymes could influence morphology was the altered phenotype, with respect to diameters and contours, of the PBP5 mutant in *E. coli* (Nelson and Young, 2000, 2001). The role of PG hydrolases in shaping bacterial morphology has major relevance in *H. pylori*, in which Csd1 and Csd2 (endopeptidases), Csd3 (a bifunctional endo- and carboxypeptidase) and Csd4 and Csd6 (carboxypeptidases) dictate helical shape (Bonis et al., 2010; Sycuro et al., 2010, 2012, 2013; Kim et al., 2014, 2015; An et al., 2015). Csd4 and Csd6, probably together with the hypothetical scaffolding protein Csd5, trim PG monomers to dipeptides, resulting in cell curvature, possibly because the trimming is localized and decreases the local availability of crosslinkable PG precursors (Sycuro et al., 2012, 2013). Csd1 and Csd2, together with the bactofilin CcmA discussed above, determine the helical twist of *H. pylori*, probably by locally cutting tetra-pentapeptide crosslinks (Sycuro et al., 2012). Csd3 appears to participate in both of these networks (Sycuro et al., 2012). Similarly, the carboxypeptidases Pgp1 and Pgp2 in *C. jejuni* are major determinants of the morphology of this bacterium (Frirdich et al., 2012, 2014; Frirdich and Gaynor, 2013).

Chemical modifications of the murein sacculus, such as amidation of the D-center of DAP in *Lactobacillus plantarum*, have also been reported to be important in cell morphology and growth (Bernard et al., 2011). The amount of PG O-acetylation affects morphology, at least in *C. jejuni* (Ha et al., 2016); an increase in PG O-acetylation caused by inactivation of the gene *ape1* leads to a significant difference in the amount and variance of curvature of these cells and a decreased colonization phenotype. However, for both examples, it is unknown whether the effects on bacterial shape and fitness are caused by the PG composition directly or by misregulation of PG-associated enzymes that are less efficient in recognizing the altered PG.

### Peptidoglycan-Independent Morphological Determinants

In addition to morphological determinants affecting the PG sacculus, PG-independent determinants are known. In some spirochetes, periplasmic flagella are responsible for the characteristic spiral or flat-wave shape (Motaleb et al., 2000) or additional twisting of the bacteria (Charon et al., 1991; Ruby et al., 1997; Picardeau et al., 2001). The periplasmic flagella deform the sacculus, which in turn deforms the flagella, resulting in the particular cell shape. This is a dynamic process that causes the bacteria to move, even in highly viscous media (Wolgemuth et al., 2006; Dombrowski et al., 2009; Harman et al., 2013). This motility is a necessary prerequisite for the virulence of the spirochete *Borrelia burgdorferi* (Sultan et al., 2013, 2015).

Another PG-independent morphological determinant is membrane composition, as demonstrated for the rod-shaped *Rhodobacter sphaeroides* (Lin et al., 2015). *R. sphaeroides* with a reduced amount of the membrane lipid cardiolipin is nearly spherical. It is not yet understood by which mechanism a reduced amount of cardiolipins leads to altered cell shape in *R. sphaeroides*. The geometry of cardiolipin molecules dictates preferential localization at sites with increased membrane curvature, notably the cell poles and the cell division site (Huang et al., 2006). Because a higher percentage of the membrane is in a curved state in spherical cells than in rod-shaped cells, one would intuitively presume that spherical cells contain more instead of less (as was the case in the *R. sphaeroides* mutant) cardiolipin. Indeed, *E. coli* minicells, in which a very high percentage of the membrane is in a highly curved state, are enriched in cardiolipin (Koppelman et al., 2001). Thus, the effect of membrane composition on cell shape might be indirect, such as by affecting the localization of lipid II or MreB, which are both linked to specific membrane organization (Ganchev et al., 2006; Strahl et al., 2014).

## Changing Cell Shape During the Life of a Bacterial Cell

Many bacterial species undergo dramatic shape changes throughout the cell cycle (dimorphic or polymorphic bacteria). Modification of the shape of sacculi might be achieved by remodulation of the spatiotemporal activation patterns of PG biosynthetic complexes and/or the frequency of cell division relative to the rate of growth. However, in many instances, shape change includes “*de novo*” differentiation of cell regions or appendages, such as “points” in *Stella vacuolata* (Vasilyeva, 1985) or prostheca in *Asticcacaulis biprosthecum* (Pate et al., 1973) and *Hyphomonas neptunium* (Leifson, 1964). These situations require additional elements that dictate when and where new complexes are assembled and activated. The recently discovered proteins from *C. crescentus* and related species (Biondi et al., 2006; Jiang et al., 2014; Persat and Gitai, 2014) are the first morphogenetic elements with such abilities. If these types of shape modifications are dependent on “sufficient and necessary” modular elements, such elements could provide excellent tools to manipulate shape in species of biotechnological interest.

### Morphological Changes throughout the Cell Cycle

The alphaproteobacterium *C. crescentus* is the best-studied bacterial model organism with a cell cycle-dependent morphology. Juvenile flagellated swarmer cells have a curved rod shape, and during development, a stalk grows from the previously flagellated cell pole. The cell eventually divides in an asymmetric fashion: the stalked mother cell can immediately undergo a new round of division, whereas the daughter cell must develop into a stalked cell before undergoing a new round of division. These cell cycle-dependent phenomena are dictated by a robust regulatory circuit that combines transcriptional and translational regulation, proteolysis, and phosphorylation (Tsokos and Laub, 2012; Woldemeskel and Goley, 2017). Cell division only occurs in the stalked cell and depends on how Z ring assembly is temporally and spatially coordinated with chromosome segregation through the actions of MipZ, CtrA, and DnaA, among other proteins (Laub et al., 2000; Thanbichler and Shapiro, 2006; Curtis and Brun, 2010). The transcriptional regulators TacA and StaR are involved in the development of the stalk (Biondi et al., 2006), but *tacA* and *staR* mutants still form stalks when starved of phosphate (Biondi et al., 2006), indicating additional regulators of stalk formation. The precise mechanisms via which the stalk is elongated remain obscure, although involvement of the elongasome components RodA and MreB (Wagner et al., 2005) and the above-mentioned bactofilins and PbpC, which localize at the base of the stalk (Kühn et al., 2010), has been demonstrated.

*Asticcacaulis* species are related to *C. crescentus* and also form stalks during their cell cycle. The location of the stalk differs between different *Asticcacaulis* species: *A. excentricus* displays one subpolar stalk, whereas *A. biprosthecum* has two bilateral stalks at midcell. These species have repurposed an ancestral regulatory protein, SpmX (Radhakrishnan et al., 2008), by adding a new domain to the C-terminus to function as a localization marker for stalk synthesis (Jiang et al., 2014). The factors recruited by SpmX for local PG synthesis for stalk production are unknown.

*Hyphomonas neptunium*, another alphaproteobacterium, is an example of a budding bacterium with a cell cycle-dependent morphology. New offspring arise from a stalk that emerges from the mother cell. As in *C. crescentus*, this cell division is asymmetric: the ovococcoid daughter cell can only divide after developing into a stalked cell itself. The cell cycle-dependent morphology of these bacteria originates from PG incorporation at specific cellular locations dependent on the stage of the cell cycle (Cserti et al., 2017). In addition, buds originate from the stalk by remodeling of the tip of the stalk. Further research is needed to understand the mechanisms that regulate and establish this morphogenetic program.

As the discussed examples show, studies of di- or polymorphic bacteria have provided a deeper understanding of the regulation and coordination of morphogenesis. As only very few bacteria with cell cycle-dependent morphologies have been investigated, many more regulatory networks will likely be discovered upon further research in this field.

### Morphological Changes Dependent on Environmental Conditions

Bacteria are strongly affected by changes in environmental conditions. Multiple species undergo morphological changes under certain conditions. These changes may be related to a transition to a metabolically inactive state or to a need to increase nutrient uptake or escape threats. Some bacteria induce a dormant state known as viable but not culturable (VBNC) upon low-temperature exposure and/or nutrient deprivation. The development of VBNC forms is associated with morphological changes in some species (Baker et al., 1983; Rollins and Colwell, 1986; Effendi and Austin, 1995; Citterio et al., 2004; Liu et al., 2017). Many Gram-negative pathogens change from rod to coccoid forms. (Barer et al., 1993). These morphological changes are in some cases correlated with regulation of the expression of cell envelope/wall genes (Asakura et al., 2007; Hung et al., 2013; Meng et al., 2015). Resuscitation of *V. parahaemolyticus* VBNC forms generates shape heterogeneity apparently caused by the increased expression of the DD-carboxypeptidase DacB (Hung et al., 2013). The morphological transition of *H. pylori* during VBNC to coccoid forms is the result of the activity of the PG hydrolase AmiA (Chaput et al., 2006, 2016), which alters PG composition to increase levels of disaccharide dipeptides (Costa et al., 1999). Remodeling of the cell wall appears to be a shared feature of VBNC induction in diverse organisms, although further research is needed to understand the relevance of this remodeling to morphogenesis.

Diverse bacteria respond to starvation conditions by forming metabolically inert spores that are smaller and often more coccoid than the cells themselves. Upon this major metabolic reprogramming (beyond simple morphological adaptation), the PG in the spores of *Bacillus* species is remodeled (Tan and Ramamurthi, 2014) to a specialized PG called the cortex. The cortex has a much lower degree of crosslinking and fewer peptide stems, with regular distribution of the atypical modification muramic δ-lactam at every second muramic acid along the PG strand (Gilmore et al., 2004). The main enzymes involved in these changes are D,D-carboxypeptidases (Popham et al., 1999) and, in the case of muramic δ-lactam, the concerted action of the amidase CwlD and the deacetylase PdaA (Gilmore et al., 2004).

Bacterial morphology and PG topology can also be influenced by bacterial growth stage. In stationary phase, the stringent response of *E. coli* governs downregulation of PG synthesis (Ishiguro and Ramey, 1976), and the sacculus undergoes a number of structural changes, including increased crosslinking (including LD-crosslinks) and reduced chain length (Pisabarro et al., 1985). *V. cholerae* follows the same dynamics but also displays RpoS-dependent cell wall chemical editing mediated by non-canonical D-amino acids (Lam et al., 2009; Cava et al., 2011). *C. crescentus* undergoes a morphological adaptation during stationary phase that causes the cells to elongate, decrease in width, and become helical (Wortinger et al., 1998). Another morphological adaptation by *C. crescentus* (and relatives such as *Asticcacaulis* species) is substantial elongation of the stalk in response to phosphate limitation (Schmidt and Stanier, 1966). This stalk elongation appears to be a strategy to either increase phosphate absorption capacity and/or elevate the cell body away from the surface (Wagner et al., 2006). The mechanisms responsible for these adaptations in *C. crescentus* remain obscure (Woldemeskel and Goley, 2017).

Upon environmental stresses, multiple bacteria (Chauhan et al., 2006; Justice et al., 2006; Stackhouse et al., 2012) increase drastically in length via a process called filamentation, which is achieved by inhibiting cell division while maintaining cell growth (Justice et al., 2008). In *E. coli*, the SOS response can trigger cell filamentation by inducing the division inhibitor SulA (Huisman and D’Ari, 1981; Bi and Lutkenhaus, 1993). In uropathogenic *E. coli* (UPEC), filamentation during urinary tract infections depends on the cell division gene *damX* (Khandige et al., 2016). UPEC undergoes additional morphological transitions in addition to filamentation, as it forms non-motile, rod-shaped intracellular bacterial communities (IBCs) (Schwartz et al., 2011). These arise upon initial invasion within the cytoplasm of bladder umbrella cells and then eventually transitions into slower-growing coccoids that form more organized biofilm-like communities (mid-IBCs). At this point, a small subset of cells further differentiates into filaments within these mid-IBCs. Eventually, the coccoid UPEC cells become motile and bacillary (late IBCs), lysing the host cell and releasing both filaments and motile rods for further rounds of invasion into neighboring bladder cells (egress and second-generation IBCs). Since each IBC represents a single invasion event, the morphological changes observed within these communities are likely part of a developmental program in which each morphotype presumably functions to facilitate intracellular growth and subsequent rounds of infection (Schwartz et al., 2011).

Many bacteria change morphology during the transition to swarming motility. This transition is induced by surface contact. Swarmer cells are characterized by increased cell length and number of flagella (Jansen et al., 2003; Kearns and Losick, 2003; Armbruster and Mobley, 2012; Partridge and Harshey, 2013). In *P. mirabilis*, PG O-acetylation decreases from 51 to 29% upon differentiation to swarmer cells (Strating et al., 2012). This differentiation is accompanied by additional changes in the PG composition as well as the autolysin profile (Strating et al., 2012).

## The Importance of Bacterial Cell Shape

Morphology affects bacterial life in multiple ways (Young, 2006). Direct evidence of roles of morphology in multiple processes has only been collected in some species. Below, we will discuss several recent studies elucidating the impact of altered morphology on multiple processes, including bacterial survival and pathogenicity.

Colonization of surfaces can be facilitated by cell shape, as shown for the curved model organism *C. crescentus* (Persat et al., 2014). *C. crescentus* mother cells use their stalk to attach to surfaces, and the daughter cells expresses pili and a flagellum at the opposite pole (Curtis and Brun, 2010). Persat et al. (2014) followed the colonization of surfaces by both curved and uncurved *C. crescentus* cells under flow in a microfluidic set-up to mimic the natural environment, i.e., freshwater lakes and streams. Curved cells formed larger and taller microcolonies than straight cells under moderate flow. The study demonstrated that in dividing cells, the daughter cell pole with the pilus is positioned closer to the surface of the microfluidic device because of the cell curvature. This positioning facilitates attachment to this same surface via retraction of the pilus and thus enhances colonization by curved cells in moderate flow.

The importance of cell shape in biofilm formation was demonstrated in a study of the alphaproteobacterium *R. sphaeroides*, which inhabits soil and anoxic water bodies (Lin et al., 2015). The wild-type rod-shaped bacteria readily form biofilms. Ellipsoidal (shortened rods) mutants and coccoid cells treated with *S-*(3,4-dichlorobenzyl)isothiourea (A22), an inhibitor of MreB, were impaired for surface attachment and biofilm formation. These results suggest that the morphological changes decrease the surface area of the bacteria in contact with the surface and neighboring cells, leading to a defect in attachment to the surface and other cells. In *Burkholderia cepacia*, an opportunistic pathogen that causes pneumonia, a spherical mutant was detected in a screening for altered biofilm formation (Huber et al., 2002). The structure of the biofilms formed by these coccoid *rodA* mutants was clearly different from those formed by rod-shaped wild-type cells and featured exceptionally thick aggregates alternating with uncolonized surface areas. These studies in *R. sphaeroides* and *B. cepacia* thus provide evidence for the relationship between morphology and biofilm formation postulated by Young (Young, 2006).

Alignment of bacterial cells is also important in some forms of social motility. *P. mirabilis* is a rod-shaped bacterium that can infect the urinary tract and can move in groups of cells aligned in parallel in a process called swarming (Schaffer and Pearson, 2015). Altering morphology by increasing the amount of bactofilin protein CcmA, expressing a truncated CcmA, or knocking out *ccmA* leads to curved *P. mirabilis* cells with inferior swarming compared with wild-type (Hay et al., 1999). This swarming defect was attributed to the inability of (irregularly) curved cells to form the neatly parallel alignment of cells required for swarming. The importance of swarming in the pathogenicity of *P. mirabilis* remains controversial: some mutants that have lost the ability to swarm are less virulent, whereas several non-motile mutants are fully virulent (Schaffer and Pearson, 2015).

The motility of single cells can also be affected by cell shape, as shown for the helical pathogens *H. pylori* and *C. jejuni* and the curved *V. cholerae*. The cell shape of these pathogens is important in colonization of the GI tract by *H. pylori* (Bonis et al., 2010; Sycuro et al., 2010, 2012), a pathogen that can cause inflammation, gastric ulcers and cancers in the human stomach (Kusters et al., 2006); *V. cholerae*, which can cause the diarrheal disease cholera; and *C. jejuni* (Frirdich et al., 2012, 2014; Stahl et al., 2016), which invades epithelial cells (Young et al., 2007). During infection, all three pathogens move through the mucus layer lining the GI tract. This environment has been simulated using gel-like substances [whose suitability for mimicking the mucus layer remains under debate (Celli et al., 2009; Yang D.C. et al., 2016)] or viscous liquids. Two straight rod-shaped mutants and two curved rod-shaped mutants of *C. jejuni* both display decreased motility in soft agar compared to wild-type (Frirdich et al., 2012, 2014; Ha et al., 2016; Stahl et al., 2016). Experiments in gel-like substances showed that the motility of some *H. pylori* mutants with altered helicity is reduced compared with wild-type (Sycuro et al., 2010, 2012), whereas other mutants do not show reduced motility (Bonis et al., 2010; Sycuro et al., 2010). Although initial studies in viscous liquids did not reveal a decrease in motility of straight *H. pylori* mutants (Sycuro et al., 2010), a recent in-depth study in a mucus-mimicking solution showed that the amount of motile cells and the median speed were both lower for non-helical mutants (Martínez et al., 2016). This finding explains the advantage of the helical cell shape for *H. pylori* during colonization by showing that helical cells are better adapted to move through the mucus layer covering the stomach epithelium. A similar process appears to occur in *V. cholerae*, in which the CrvA-driven curvature promotes motility in hydrogels and confers an advantage in host colonization and pathogenesis (Bartlett et al., 2017). In *C. jejuni*, rod-shaped mutants colonize the lumen in a mouse infection model, whereas straight-rod mutants do not cross the mucus layer to infect the intestinal crypts and therefore are not pathogenic (Stahl et al., 2016). In addition, straight *C. jejuni* mutants are also less capable of forming biofilms; biofilm formation has been linked to cell survival under stressful conditions (Murphy et al., 2006; Reuter et al., 2010). Further research might demonstrate whether this decreased biofilm-forming capacity has any relevance for the survival of *C. jejuni* inside or outside its host.

Cell shape might facilitate reduced detection of bacteria by the immune system (Veyrier et al., 2015). The opportunistic pathogens *Neisseria meningitidis* and *Moraxella catarrhalis* are both adapted to live in the human nasopharynx. Both species are coccoid, but their ancestors were rod-shaped and ovococcoid, respectively. A recent study showed that loss of the gene *yacF*, which encodes a protein involved in the transition from elongation to division, could explain the morphological evolution of both species (Veyrier et al., 2015). In addition to this morphological change, the amount of pentapeptides is increased in the PG of *N. meningitidis* cocci. These PG sacculi are recognized less efficiently by the Nod1 and Nod2 receptors of the innate immune system. Veyrier et al. (2015) hypothesized that the smaller cell surface of the coccoid bacteria might also reduce attacks from the immune system. The entire cell surface of the coccoid cells is covered in pili, whereas rod-shaped cells display pili on the poles only. Thus, coccoid cells might attach to the nasopharyngeal mucosa more efficiently.

Morphology might also play a role in the infection process itself. *Shigella flexneri* is a rod-shaped pathogen that infects epithelial cells in the large intestine in a multi-step process in which its type III secretion system (T3SS) plays an important role (Schroeder and Hilbi, 2008). Although A22-treated coccoid cells still attach to eukaryotic cells *in vitro*, invasion of these cells is clearly impaired (Noguchi et al., 2008). Furthermore, the coccoid cells secrete less T3SS effector proteins, suggesting that the altered morphology leads to a decrease in effector secretion through T3SS, possibly due to mislocalization of the T3SS proteins. Further research is needed to establish if the reduced pathogenicity is directly caused by the altered morphology or (in)directly by inactivation of the MreB cytoskeleton. Indeed, in the enteric pathogen *Salmonella typhimurium*, inactivation of the MreB cytoskeleton by depletion of the accessory proteins MreC and MreD leads to spherical cells that are impaired in the disruption of epithelial tight junctions *in vitro* and colonization in a mouse model (Bulmer et al., 2012). In this case, however, the altered morphology plays only a minor role, if any, in the attenuated virulence. Instead, the disruption of the MreB cytoskeleton leads to downregulation of genes involved in pathogenicity (Bulmer et al., 2012; Doble et al., 2012).

Moreover, certain bacteria change shape as a strategy to boost survival when confronting environmental stresses, as discussed in the previous section. Filamentation protects UPEC cells from phagocytosis by neutrophils during infection of the bladder (Justice et al., 2004, 2006; Horvath et al., 2011). *Legionella pneumophila* filamentous cells are less easily engulfed by phagocytes (Prashar et al., 2012, 2013). In *Haemophilus influenza* causing otitis, the influence of filamentous cells on biofilm architecture increases the persistence of the pathogen in an animal model (Szelestey et al., 2013). Filamentous cells of *S. enterica* (Humphrey et al., 2011) and *Edwardsiella tarda* (Wang et al., 2014) are less able to invade epithelial cells. Filamentation is also used by multiple bacteria as a strategy to escape predation by protists (Güde, 1979; Hahn et al., 1999; Corno and Jürgens, 2006), although in some cases filamentation can’t prevent bacteria from being eaten (Wu et al., 2004).

## Morphological Determinants as Targets for Antimicrobials

The rise of antibiotic resistance in pathogenic bacteria and the very limited success in developing new antibiotics drives the search for novel targets in antimicrobial research (Spellberg et al., 2008; Fishbach and Walsh, 2009). The majority of antibiotics currently in use as well as those under development (Boucher et al., 2013) act on membrane stability, PG biosynthesis, folate biosynthesis, DNA replication, transcription and translation (Hurley et al., 2016). Strategies under investigation include hijacking toxin-antitoxin systems (Chan et al., 2015), inhibiting bacterial cell division (Lock and Harry, 2008; Hurley et al., 2016) and blocking the T3SS to render bacteria non-pathogenic (McShan and De Guzman, 2015).

As exemplified by this last strategy, it might be possible to develop antimicrobials that are not bactericidal *per se* but that target the ability of bacteria to cause unwanted effects, such as disease. A meta-analysis showed that the curing efficiencies of bacteriostatic and bactericidal antibiotics are equivalent (Nemeth et al., 2015), supporting the validity of this strategy to address infectious diseases. However, the use of bacteriostatic antibiotics might increase the incidence of antibiotic resistance as living bacteria can mutate and develop resistance, whereas dead bacteria cannot (Stratton, 2003). However, if the targeted pathways are of key importance for bacterial survival or proliferation, it is expected that the bacteria will eventually die, even if the drugs are mechanistically bacteriostatic. This indeed appears to be the case for antibiotics targeting cell division (Lock and Harry, 2008).

As bacterial cell shape can impact the ability of bacteria to survive in their specific niche as well as colonize hosts, escape the immune system and cause disease, shape might be a good target for antimicrobials. The exploration of morphological determinants of bacteria as drug targets has been limited and has focused primarily on bacterial cytoskeletal elements (Vollmer, 2006). In the following paragraphs, we will discuss the potential indications for morphology inhibitors and the possible advantages and disadvantages of morphology as a target for inhibitors. Furthermore, we will introduce examples of inhibitors currently under development and outline possibilities for future research directions.

### Possible Applications of Inhibitors of Bacterial Cell Shape

Multiple applications might benefit from inhibitors of bacterial morphology. The most obvious use would be to make pathogenic bacteria unfit to colonize their host, escape the immune system and cause disease. This strategy will only be effective if the morphology of the bacterium is important for survival or virulence in the host. The most obvious host is humans, but this strategy is also applicable to plant or animal species, such as food crops or endangered species. Indeed, antibiotics are commonly used in agriculture, where antibiotic resistance is also a problem (Thanner et al., 2016).

Another possible application is inhibition of biofilm formation. Bacterial biofilms are a substantial problem in the food and beverage industries (Brooks and Flint, 2008), medicine (Francolini and Donelli, 2010) and water treatment (Nguyen et al., 2012). As discussed above, bacterial morphology has been shown to influence biofilm formation (Huber et al., 2002; Lin et al., 2015), and therefore targeting cell shape might be a very useful strategy to combat biofilm formation.

Furthermore, it might be promising to use shape inhibitors to change the characteristics of bacteria to increase their suitability for biotechnological applications. Cell shape dictates the ratio between the membrane area and volume of a bacterial cell, a parameter that affects both substrate uptake (Schulz and Jørgensen, 2001) and product excretion. If bacteria are used to degrade certain substances, the uptake efficiency of the bacteria is of high interest. The ability of bacteria to efficiently export proteins or other substances is crucial in many biotechnological applications.

### Targeting Morphological Determinants: Advantages and Disadvantages

An important advantage of morphological determinants as drug targets is the potential for broad-spectrum as well as narrow-spectrum antibiotics. Successfully targeting widespread determinants, such as MreB, will lead to broad-spectrum antibiotics. Other determinants, such as the PG hydrolases that shape *H. pylori* (Sycuro et al., 2012) and *C. jejuni* (Frirdich et al., 2012; Stahl et al., 2016), are only conserved in certain bacteria, in this case, several helical or curved delta- and epsilonproteobacteria (Sycuro et al., 2012). Targeting these determinants thus allows for drugs against a specific class of pathogens. In addition to the morphological determinants themselves, the interactions of these proteins with other macromolecules can possibly be used as targets (Zoraghi and Reiner, 2013). This strategy can be used to tune the specificity of these drugs. For example, bacterial IF-like filaments such as bactofilins are widespread, but their interaction partners appear to vary. Therefore, targeting the site of interaction between bactofilins and the interacting proteins in complexes responsible for a certain morphology might be an excellent strategy to obtain very specific antimicrobials. As research into the morphological determinants of various bacteria proceeds, it is probable that multiple new potential drug targets will be identified, of which the majority are likely to be rather specific.

Why would it be relevant to develop both broad-range and narrow-range antibiotics? With respect to the curative success rate, broad-spectrum and pathogen-directed antibiotic treatments show very similar efficiency, at least in pneumonia (van der Eerden et al., 2005; Williams et al., 2013). In the case of serious bacterial infections requiring immediate treatment, broad-spectrum antibiotics are the drugs of choice because they enable treatment to start before the pathogen is identified (Kollef, 2008). However, the use of broad-spectrum antibiotics also poses problems. Broad-spectrum antibiotics kill bacteria other than pathogens (Rea et al., 2011), including beneficial microbiome species (Brown and Wright, 2005), creating a more favorable environment for antibiotic-resistant bacteria (Harbarth et al., 2002). When the pathogen is known (although routine identification would require considerable effort from the healthcare system), the use of narrow-range antibiotics is favorable because of the reduced collateral damage to other microbiome species (Rea et al., 2011) and the reduced risk of secondary infections (Palmer et al., 1995).

Another advantage of morphological determinants over some other possible targetable pathways is that most are specific to prokaryotes, thus reducing the likelihood of toxicity to the host due to binding to eukaryotic proteins with structures similar to the bacterial targets.

As discussed above, a potential disadvantage of morphological determinants as drug targets is that most of these drugs might not be bactericidal, facilitating development of resistance. Further research on different inhibitors at relevant concentrations and in different species is required; in some cases, the negative effect of the alteration of morphology might ultimately result in bacterial death. More research is also needed to establish whether changing bacterial morphology leads to unwanted side effects, such as reduced detectability or less efficient clearing by the immune system. The use of these inhibitors will probably impact microbial community composition in unexpected ways since inhibiting the target species might free up niches for other bacteria, including pathogens or biofilm-forming bacteria.

### Existing Inhibitors Targeting Morphological Determinants

Multiple inhibitors targeting FtsZ have been described (Hurley et al., 2016), but these inhibitors impact cell division more than cell shape *per se* and will therefore be discussed only briefly. The six known classes of FtsZ inhibitors function via either decreasing or increasing the GTPase activity of FtsZ (Hurley et al., 2016) or altering the interactions between monomers or protofilaments (Vollmer, 2006). Other cell-division inhibitors target the interaction between FtsZ and its membrane anchor ZipA (Sutherland et al., 2003; Jennings et al., 2004a,b; Tsao et al., 2006) or stimulate uncontrolled proteolysis of FtsZ by the protease ClpP (Sass et al., 2011).

Several inhibitors targeting MreB derived from both chemical synthesis (Iwai et al., 2002; Robertson et al., 2007; Takacs et al., 2010) and natural sources (Rodríguez et al., 2008; Molshanski-Mor et al., 2014) have been described. The two most common inhibitors, *S-*(3,4-dichlorobenzyl)isothiourea (A22) and its derivative *S-*(4-chlorobenzyl)isothiourea (MP265), bind close to but not in the nucleotide-binding site of MreB (van den Ent et al., 2014). As illustrated in recent molecular dynamics studies, binding of these inhibitors leads to slower release of γ-phosphate upon ATP hydrolysis (Awuni et al., 2016). Inhibitor-bound MreB can still polymerize in an ATP-dependent fashion (Robertson et al., 2007), but its dimerization into stable double protofilaments is hindered (van den Ent et al., 2014). The indole-class inhibitor CBR-4830 binds in the nucleotide-binding site and prevents ATP-dependent polymerization (Robertson et al., 2007). No detailed mechanistic understanding of the inhibition process is available for the other MreB inhibitors, although the binding site of the T7 phage gene product 0.6 on MreB is known (Molshanski-Mor et al., 2014). Strikingly few studies have investigated the toxicity of MreB inhibitors against eukaryotic cells, even though this is an obvious prerequisite for the development of MreB inhibitors into antibiotics. The only study that has been performed suggests that the inhibitor A22 is cytotoxic and genotoxic to human blood cells at concentrations exceeding 4.3 μM (Bonez et al., 2016), which is lower than the minimal inhibitory concentration (MIC) for multiple bacterial species (Foss et al., 2011). In addition, the structurally similar *S-*benzylisothiourea was shown to be toxic to rats (Shirota et al., 1997).

A phosphonic acid-based pseudopeptide inhibitor of the PG hydrolases Csd4 and Pgp1, which are required for the helical cell shapes of *H. pylori* and *C. jejuni*, respectively, was recently developed via targeted drug design (Liu et al., 2016). The pseudopeptide binds in the active site of the enzyme and mimics an intermediate stage in the cleavage of mesoDAP from the uncrosslinked PG stem peptide (Liu et al., 2016). The inhibitor can cross the outer membrane to induce cell straightening of both *H. pylori* and, albeit with lower efficiency due to the polysaccharide capsule, *C. jejuni* (Liu et al., 2016).

### Future Perspectives for Drugs Targeting Morphological Determinants

The availability of several inhibitors targeting morphological determinants is a good first step toward the development of drugs for use in the clinic. However, extensive work remains, and it is unclear if pharmaceutical companies find drugs targeting morphology sufficiently promising for investment. Many of the potential shape-targeting drugs are expected to be narrow-range antibiotics. Developing new antibiotics is very expensive, and pharmaceutical companies are unlikely to invest considerable resources in developing drugs with a very limited market (Walsh, 2003). Initial screening for lead structures with shape-inhibiting action has always been very tedious, requiring screening by manual microscopy, further adding to the unattractiveness of these inhibitors to companies developing antimicrobials. This problem has recently been solved by the application of automated microscopy and image analysis (Choi et al., 2014) and flow cytometry cell sorting (Laubacher et al., 2013; Sycuro et al., 2013) to screen for bacterial morphology, enabling high-throughput, low-cost screening of large compound libraries for effects on bacterial morphology.

Further research by the academic community might aid further investigation of the drug potential of inhibitors targeting morphological determinants. This research should focus on multiple goals. First, the importance of shape in bacterial survival (in the environment as well as in the host) and virulence should be studied in many more bacteria. Much more research is also needed to identify additional morphological determinants and molecular mechanisms underlying cell shape, particularly in (pathogenic) bacteria with non-standard cell shapes [as eloquently proposed elsewhere (Kysela et al., 2016)]. These two research lines will form the basis for the development of novel inhibitors via targeted design or high-throughput screening of compound libraries.

In parallel, more research is needed on existing inhibitors. These endeavors should focus on investigating the effects of these inhibitors on additional species and establishing their toxicity in eukaryotic hosts. Further elucidation of the working mechanism of these inhibitors might enable targeted optimization to develop next-generation inhibitors that are effective at lower (more practical) doses. Studies of the occurrence of resistant strains could provide information on whether these inhibitors represent good drugs alone or in combination with drugs that suppress resistance through elevated efflux.

Morphological determination continues to be an important field of fundamental research in which many open questions remain. The development of several inhibitors demonstrates the need for further study and might ultimately lead to drugs targeting bacterial morphology to control bacterial survival and virulence.

## Author Contributions

All authors listed have made a substantial, direct and intellectual contribution to the work, and approved it for publication.

## Conflict of Interest Statement

The authors declare that the research was conducted in the absence of any commercial or financial relationships that could be construed as a potential conflict of interest.
